# Difference in Postural Control during Quiet Standing between Young Children and Adults: Assessment with Center of Mass Acceleration

**DOI:** 10.1371/journal.pone.0140235

**Published:** 2015-10-08

**Authors:** Naoko Oba, Shun Sasagawa, Akio Yamamoto, Kimitaka Nakazawa

**Affiliations:** 1 Department of Life Sciences, Graduate School of Arts and Sciences, The University of Tokyo, Komaba, Meguro-ku, Tokyo, Japan; 2 Research Institute of Physical Fitness, Japan Women’s College of Physical Education, Kita-karasuyama, Setagaya-ku, Tokyo, Japan; Ludwig-Maximilian University, GERMANY

## Abstract

The development of upright postural control has often been investigated using time series of center of foot pressure (COP), which is proportional to the ankle joint torque (i.e., the motor output of a single joint). However, the center of body mass acceleration (COM_acc_), which can reflect joint motions throughout the body as well as multi-joint coordination, is useful for the assessment of the postural control strategy at the whole-body level. The purpose of the present study was to investigate children’s postural control during quiet standing by using the COM_acc_. Ten healthy children and 15 healthy young adults were instructed to stand upright quietly on a force platform with their eyes open or closed. The COM_acc_ as well as the COP in the anterior–posterior direction was obtained from ground reaction force measurement. We found that both the COM_acc_ and COP could clearly distinguish the difference between age groups and visual conditions. We also found that the sway frequency of COM_acc_ in children was higher than that in adults, for which differences in biomechanical and/or neural factors between age groups may be responsible. Our results imply that the COM_acc_ can be an alternative force platform measure for assessing developmental changes in upright postural control.

## Introduction

Upright postural control is essential for activities of daily living. It takes almost a year for a human infant to achieve an independent bipedal stance, and thereafter around a dozen years to develop adult-like postural control [1–3]. The development of upright postural control during quiet standing has often been investigated by quantifying spontaneous postural sway in the time and/or frequency domains [4–9]. These previous studies have reported that the amplitude [4,6,7], area [9], speed (i.e., total path length divided by the trial duration) [5–9], and frequency [4,8] of the sway decrease with age from 12 months old to 15 years old.

However, it should be noted that all studies mentioned above used center of foot pressure (COP) analyses to assess postural control. According to an inverse dynamics calculation, the COP position relative to the ankle joint axis is proportional to the ankle joint torque [10]. Because the ankle joint plays a primary role in stabilizing quiet standing, these studies based on COP analysis can be useful for shedding light on some fundamental aspects of postural control. In contrast, several recent studies have shown that joints above the ankle (e.g., the hip and knee) also play an important role in controlling the center of body mass (COM) even in an unperturbed situation, and have suggested that human quiet standing is a multi-joint motor task [11–17]. Furthermore, regarding the development of upright postural control, Wu et al. [18] demonstrated that it is accompanied by the alteration of multi-joint coordination. Given these observations, it is important to investigate postural control at the whole-body level to illuminate the postural control strategy adopted by the central nervous system and its development in children. Note that, in this paper, we use “strategy” to mean kinematic/kinetic coordination among the joints (see next paragraph for details), which is different from the conventional use of “strategy” (i.e., ankle/hip strategy [19]).

Some recent studies have suggested an alternative measure of spontaneous postural sway [20,21]: the translational acceleration of the COM (COM_acc_), which can be obtained by force platform measurement as easily as the COP by dividing horizontal ground reaction force (GRF) by body mass. The COM_acc_ is a linear summation of joint angular accelerations [11,13,22]. Furthermore, the angular acceleration of each joint is induced by torques of all joints throughout the body [23]. Therefore, the COM_acc_ is supposed to serve as a whole body measure that can reflect coordination and dynamic interaction among the joints. Recently, Masani et al. [20] and Yu et al. [21] have demonstrated that the COM_acc_ is very sensitive to age- and/or disease-related changes in the postural control system during quiet standing.

If multi-joint coordination during quiet standing changes with age from children to adults [18], then the development of postural control can be well captured by this whole-body measure. Therefore, the purpose of the present study was to test the hypothesis that the COM_acc_ can clearly distinguish the difference in postural control during quiet standing between young children and young adults.

## Material and Methods

### Participants

Twenty-five healthy children (17 girls and 8 boys) aged 3–6 years old with no known developmental delays (mean ± SD: age 4.8 ± 0.8 years old, height 108.9 ± 5.8 cm, and mass 17.9 ± 2.3 kg) and 15 healthy young adults (6 females and 9 males, age 25.7 ± 2.2 years old, height 167.0 ± 9.5 cm, and mass 60.9 ± 10.7 kg) participated in this study. They had no history of neurological disorders. All participants gave written informed consent according to the principles of the Declaration of Helsinki, which was approved by the Committee on Human Experimentation at the Graduate School of Arts and Sciences, The University of Tokyo.

### Experimental task

Participants were instructed to stand upright quietly on a force platform (Type 9281B, Kistler, Winterthur, Switzerland) for 30 s with their eyes open (EO) or closed (EC). They stood barefoot with their arms hanging along the sides of their body, and their feet were parallel and a shoulder-width apart. Three trials were performed for each visual condition. Because some of the 3- and 4-year-old children could not understand and accomplish the postural task required by the experimenter, the data of 5- and 6-year-old children (6 girls and 4 boys; age 5.4 ± 0.5 years old, height 111.8 ± 5.3 cm, and mass 18.7 ± 2.5 kg) are analyzed hereafter.

### Data collection

The GRFs in the vertical and anterior–posterior directions were measured by the force platform. All data were sampled at 1 kHz using a 16-bit analog-to-digital converter (PowerLab, ADInstruments, Bella Vista, NSW, Australia), and then downsampled at 100 Hz. From the GRF data, the COP position (COP) and the COM_acc_ were calculated. The COM_acc_ was obtained by dividing the GRF in the anterior–posterior direction by the participants’ body mass (except the feet) [13,17,20]. Both the COP and COM_acc_ data were low-pass filtered at a frequency of 10 Hz using a fourth-order Butterworth filter with zero phase-lag [20]. For all signals, we selected the data from a 28-s period in the middle portion of the collected data for further analyses.

### Data analysis

The standard deviations (SDs) of the COP and COM_acc_ were calculated for the time domain analysis. For the frequency domain analysis, the power spectral density function (PSD) was computed using Welch’s method (Matlab function *‘pwelch’*, Mathworks, Natick, MA, USA). The COM_acc_ signal in each trial was divided into four segments of 10 s (1000 points). It should be noted that 50% (500 points) of the data were overlapped with adjacent segments. A 1000-point fast Fourier transform algorithm was applied to each segment to yield the power spectrum after being passed through a Hamming window. The power spectrum of each segment was ensemble-averaged into the PSD for a single trial. This frequency domain analysis procedure was similar to those used in the relevant literature [11,23–25]. The averaged PSD of the COM_acc_ over three trials was smoothed using a seven-point moving average technique. Then, the mean power frequency (MPF) for the 0- to 10-Hz bandwidth was calculated as follows:

$$\text{MPF}=\int_{0}^{10}f\cdot P(f)df/\int_{0}^{10}P(f)df$$

where *f* is the frequency and *P*(*f*) is the PSD. The maximum value of the power was determined as the peak power of the COM_acc_. The frequency at which the peak power of the COM_acc_ was observed was defined as the peak power frequency (PPF).

### Statistical analysis

Because a significant difference in the participants’ body heights between children and adults was found (*P* = 2.76 × 10^−14^, unpaired Student’s t-test), to enable comparisons across data by accounting for height differences, we performed normalizations to yield dimensionless quantities. Namely, the SDs of the COP and COM_acc_ were divided by the body height, and the peak power of the COM_acc_ was divided by the squared body height. The effects of gender, age, and visual condition were analyzed using three-way analysis of variance with repeated measures. The level of statistical significance was set at *P* < 0.05. When a significant interaction between age group and visual condition was found, a paired Student’s t-test was performed for comparison between visual conditions, and an unpaired Student’s t-test for equal variance and an unpaired Welch’s test for unequal variance were performed for comparison between children and adults. Thereafter, Holm’s correction for multiple tests was performed, and only corrected *P* < 0.05 results were considered to be significant. To examine visual dependency in postural control, the rate of increase from the EO to the EC condition (i.e., Romberg’s quotient) was calculated for each measure and compared between age groups using an unpaired Student’s t-test for equal variance and an unpaired Welch’s test for unequal variance.

## Results

### Time domain analysis of COP and COM_acc_

**Fig 1:**
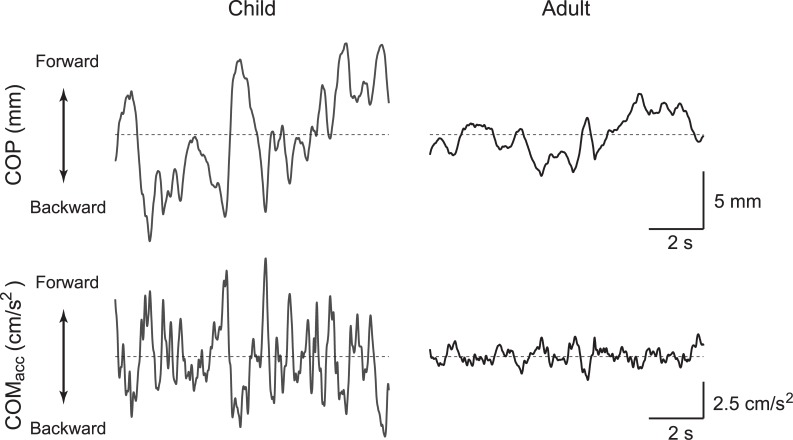
Representative examples of time series of center of foot pressure (COP) and center of mass acceleration (COM_acc_) during quiet standing. Representative examples of COP (*top panel*) and COM_acc_ (*bottom panel*) for a child (*left panel*) and an adult (*right panel*) in the eyes open condition. *Horizontal dashed lines* in the top and bottom panels indicate mean position of COP and COM_acc_ = 0, respectively.

Fig 1 illustrates examples of COP (*top panel*) and COM_acc_ (*bottom panel*) time series for a child (*left panel*) and an adult (*right panel*) in the EO condition. Note that only 10 s of data from the 30-s trial are presented in this figure to emphasize the signal features. The amplitude of the COP fluctuation in the child appears to be larger than that in the adult. Similarly, the amplitude of the COM_acc_ fluctuation was observed to be much larger in the child.

**Fig 2:**
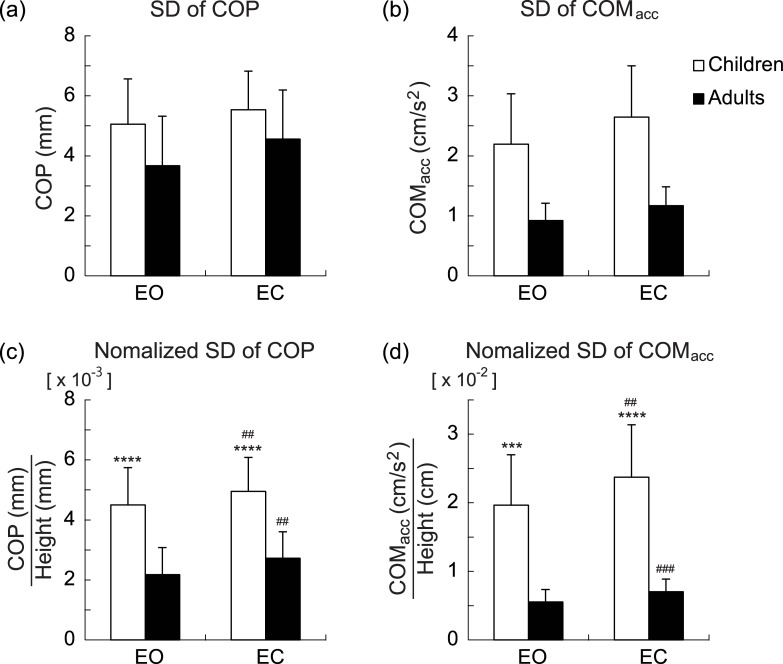
Group mean values of SD of center of foot pressure (COP), and center of mass acceleration (COM_acc_). Group mean values of unnormalized SD of COP (*a*) and COM_acc_ (*b*) for each age group and visual condition, and normalized COP (*c*) and COM_acc_ (*d*). *White* and *black bars* indicate child and adult groups, respectively. Data are group means ± SD. *** and **** indicate statistical significances of *P* < 0.001 and *P* < 0.0001, respectively, between age groups. ## and ### indicate statistical significances of *P* < 0.01 and *P* < 0.001, respectively, between visual conditions.

Fig 2 shows group mean values of unnormalized SDs of the COP (*a*) and COM_acc_ (*b*), and normalized SDs of the COP (*c*) and COM_acc_ (*d*) for each age group and visual condition. For the normalized COP, the main effects of age group and visual condition were significant (*F*(1, 21) = 33.069, *P* = 1.57 × 10^−5^; *F*(1, 21) = 8.678, *P* = 0.008, respectively). The main effect of gender was not significant, and nor was any interaction (main effect of gender: *F*(1, 21) = 0.210, *P* = 0.652; visual condition × age group interaction: *F*(1, 21) = 0.191, *P* = 0.667; visual condition × gender interaction: *F*(1, 21) = 0.692, *P* = 0.415; age group × gender interaction: *F*(1, 21) = 0.010, *P* = 0.920; visual condition × age group × gender: *F*(1, 21) = 0.616, *P* = 0.441). For the normalized COM_acc_, the main effects of age group and visual condition were both significant (*F*(1, 21) = 55.559, *P* = 2.51 × 10^−7^; *F*(1, 21) = 54.049, *P* = 3.10 × 10^−7^, respectively). The main effect of gender was not significant, and nor was any interaction except for between visual condition and age group (main effect of gender: *F*(1, 21) = 0.040, *P* = 0.842; visual condition × gender interaction: *F*(1, 21) = 0.780, *P* = 0.387; age group × gender interaction: *F*(1, 21) = 0.537, *P* = 0.472; visual condition × age group × gender interaction: *F*(1, 21) = 0.072, *P* = 0.790). Because the interaction between age group and visual condition was significant (*F*(1, 21) = 10.883, *P* = 0.003), the simple effects were examined for each age group and visual condition. An unpaired Welch’s test with Holm’s correction indicated that, in both visual conditions, the SD of the COM_acc_ in children was significantly larger than that in adults (EO: *P* = 3.14 × 10^−4^, EC: *P* = 5.62 × 10^−5^). Also, a paired Student’s t-test with Holm’s correction revealed that the SD of the COM_acc_ in the EC condition was significantly larger than that in the EO condition in both age groups (children: *P* = 0.002, adults: *P* = 6.75 × 10^−4^). Taking these simple effects together, the significant interaction indicates that the increase in the COM_acc_ SD with eyes closed was significantly larger in children than in adults. However, because the SDs of the COM_acc_ in children under both visual conditions were about four times greater than those of adults (Fig 2d), we cannot directly infer visual dependency from this interaction effect. To enable comparison of visual dependency between age groups, we then calculated the rate of increase of the COM_acc_ SD from the EO to the EC condition. An unpaired Student’s t-test revealed that there was no significant difference in the EC/EO ratio between children (1.2 ± 0.2) and adults (1.3 ± 0.3, *P* = 0.443).

### Frequency domain analysis of COM_acc_

**Fig 3:**
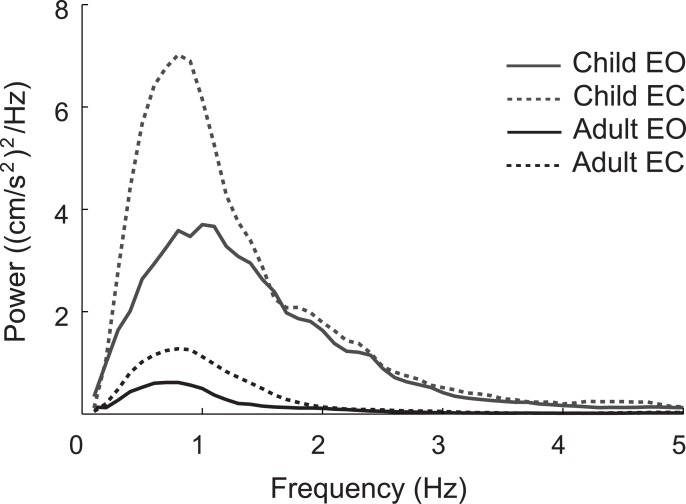
Representative power spectral density functions of center of mass acceleration (COM_acc_). Representative power spectral density functions of COM_acc_ in the eyes open (EO, *solid lines*) and eyes closed (EC, *dashed lines*) conditions for the same participants as shown in Fig 1. *Gray* and *black colors* indicate the results for the child and adult participants, respectively.

**Fig 4:**
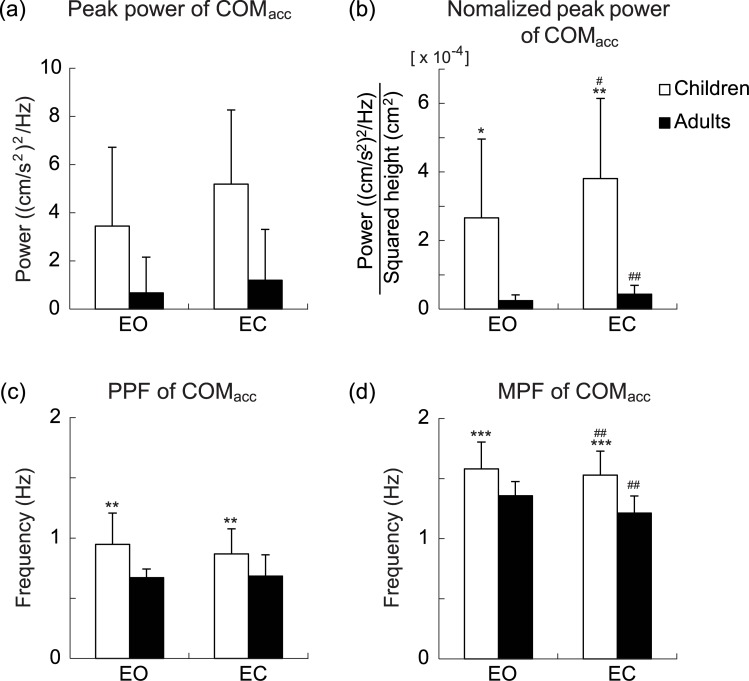
Group mean values of the peak power, peak power frequency (PPF), and mean power frequency (MPF) of the center of mass acceleration (COM_acc_). Group mean values of the unnormalized peak power (*a*), normalized peak power (*b*), peak power frequency (*c*) and mean power frequency (*d*) of the COM_acc_ for each age group and visual condition. *White* and *black bars* indicate the results for child and adult groups, respectively. Data are group means ± SD. *, **, and *** indicate statistical significances of *P* < 0.05, *P* < 0.01, and *P* < 0.001, respectively, between age groups. # and ## indicate statistical significance of *P* < 0.05 and *P* < 0.01, respectively, between visual conditions.

Fig 3 illustrates representative PSDs of the COM_acc_ in the EO (*solid lines*) and EC (*dashed lines*) conditions for the same participants as shown in Fig 1 (child: *gray line*; adult: *black line*). For all frequencies, the spectral power of the COM_acc_ was much larger in the child than in the adult. Moreover, the child showed a peak power at a higher frequency than the adult. Fig 4 summarizes the unnormalized peak power (*a*), the normalized peak power (*b*), the peak power frequency (PPF, *c*), and mean power frequency (MPF, *d*) of the COM_acc_ for each age group and visual condition. For the normalized peak power of the COM_acc_, significant main effects of age group and visual condition was observed (*F*(1, 21) = 22.611, *P* = 1.07 × 10^−4^ and *F*(1, 21) = 25.964, *P* = 4.79 × 10^−5^, respectively). The main effect of gender was not significant, nor was any interaction except for between visual condition and age group (main effect of gender: *F*(1, 21) = 0.102, *P* = 0.753; visual condition × gender interaction: *F*(1, 21) = 2.285, *P* = 0.145; age group × gender interaction: *F*(1, 21) = 0.314, *P* = 0.581; visual condition × age group × gender interaction: *F*(1, 21) = 1.193, *P* = 0.287). There was a significant interaction between age group and visual condition (*F*(1, 21) = 12.397, *P* = 0.002). An unpaired Welch’s test with Holm’s correction showed that the peak power of the COM_acc_ in children was significantly greater than that in adults in both visual conditions (EO: *P* = 0.035, EC: *P* = 0.003). Also, a paired Student’s t-test with Holm’s correction revealed that the peak power of the COM_acc_ was significantly greater in the EC condition than in the EO condition in both age groups (children: *P* = 0.013, adults: *P* = 4.71 × 10^−4^). As well as in the case of the COM_acc_ SD, an unpaired Welch’s test showed no significant difference in the EC/EO ratio of the peak power between children (1.7 ± 0.5) and adults (2.0 ± 1.1, *P* = 0.269).

For the PPF of the COM_acc_, the main effect of age group was significant (*F*(1, 21) = 12.699, *P* = 0.002). The main effects of visual condition and gender were not significant and neither was any interaction (main effect of visual condition: *F*(1, 21) = 0.432, *P* = 0.518; main effect of gender: *F*(1, 21) = 0.278, *P* = 0.603; visual condition × age group interaction: *F*(1, 21) = 0.516, *P* = 0.481; visual condition × gender interaction: *F*(1, 21) = 2.381, *P* = 0.138; age group × gender interaction: *F*(1, 21) = 0.326, *P* = 0.574; visual condition × age group × gender interaction: *F*(1, 21) = 0.146, *P* = 0.706). For the MPF of the COM_acc_, the main effects of age group and visual condition were both significant (*F*(1, 21) = 19.295, *P* = 2.54 × 10^−4^ and *F*(1, 21) = 8.343, *P* = 0.009 for age group and visual condition, respectively). The main effect of gender was not significant, nor was any interaction (main effect of gender: *F*(1, 21) = 0.251, *P* = 0.621; visual condition × age group interaction: *F*(1, 21) = 2.277, *P* = 0.146; visual condition × gender interaction: *F*(1, 21) = 0.431, *P* = 0.519; age group × gender interaction: *F*(1, 21) = 0.576, *P* = 0.456; visual condition × age group × gender: *F*(1, 21) = 0.007, *P* = 0.933).

## Discussion

In the present study, we investigated the difference in the postural control during quiet standing between children and adults using COM_acc_, which can assess the postural control strategy at the whole-body level. The present results showed that both the COM_acc_ and COP can distinguish the difference between age groups and visual conditions (Fig 2). As previously noted, the COP position relative to the ankle joint axis is proportional to the amplitude of the ankle joint torque exerted at that moment. That is to say, the COP time series reflects fluctuations in the motor output of one primary joint. Conversely, the COM_acc_, which is a linear summation of the joint angular accelerations, can reflect the postural control strategy throughout the body (i.e., the motor output of all joints and multi-joint coordination). The current results are in agreement with previous studies indicating the high sensitivity of the COM_acc_ in assessing postural control. For example, Masani and colleagues [20,21] demonstrated that COM_acc_ is very sensitive to age- and disease-related changes in the postural control system. Corriveau et al. [26,27] have also reported that a closely related measure, COP−COM, which is usually proportional to the COM_acc_ during quiet standing [10,20,21], is an effective variable for the detection of postural instability in older people with diabetic neuropathy or following stroke.

Although a significant interaction between age group and visual condition was found for the SD and peak power of the COM_acc_, there were no significant differences in the EC/EO ratios of those measures between age groups. These results, suggesting no significant difference in the visual dependency between age groups, are not consistent with the idea that children are more vision-dependent than adults during dynamic balance tasks [28,29].

The frequency domain analysis revealed the MPF of the COM_acc_ to be 1.6 ± 0.2 Hz and 1.3 ± 0.2 Hz for children and adults, respectively (note that MPFs reported here are the mean values of both visual conditions). The MPF of COM_acc_ in adults is in complete agreement with the MPF of soleus and gastrocnemius length adjustments during quiet standing (1.3 Hz) demonstrated by Loram et al. [30]. Furthermore, the subsequent statistical analysis indicated that the PPF and MPF of the COM_acc_ were significantly higher in children than in adults (Fig 4b and 4c). As possible explanations for the differences in the PPF and MPF between the age groups, biomechanical and/or neural factors can be proposed. One of the former would be the difference in the inertial properties of the body segments between the age groups. We know for a fact that we can easily balance an upright broom (i.e., an adult’s body) on the palm and that we find it very difficult to do this with a pencil (i.e., a child’s body) [31]. This is because the relatively small inertia of the pencil requires us to balance it with a shorter time constant. In other words, more frequent adjustments in hand position are needed to balance the pencil successfully. Another biomechanical factor would be the number of the joints involved in upright postural control. For example, Günther et al. [32] estimated the eigenfrequency of the single-link inverted pendulum model of quiet standing to be approximately 0.2 Hz. For a non-inverted version of the triple-link pendulum model, however, the ankle, knee, and hip eigenmovements were calculated to have eigenfrequencies of 0.48, 1.13, and 3.47 Hz, respectively [12]. If children behave more like a multi-joint inverted pendulum during quiet standing [18], these higher eigenfrequencies are expected to dominate the frequency spectrum of the COM_acc_ and thereby to increase the MPF. For the neural factors, it is possible that the relative contributions of different neuronal loops (i.e., short and long loops) to postural control differ between children and adults. It has been demonstrated, from COP-based measurements, that body sway below 0.5–1.0 Hz is related to visual-vestibular information (long loop), whereas sway above 0.5–1.0 Hz is associated with somatosensory information (short loop) [33,34].

In both age groups, the MPF of the COM_acc_ was lower in the EC condition than in the EO condition (Fig 4b). Given that the amplitude of the COM_acc_ was larger in the EC condition, the lower MPF in the EC condition may be attributable to an increased power of the COM_acc_ in the low-frequency band.

To date, developmental change in upright postural control has usually been investigated using the COP, which is an indicator of motor output from the ankle joint. Although the ankle joint has a crucial role in stabilizing quiet standing, recent studies have shown that proximal joints (e.g., knee and hip) also have a substantial contribution to the balancing act [11–17]. Furthermore, Wu et al. [18] demonstrated, using an uncontrolled manifold approach [35], that even in children the COM during quiet standing is controlled by the motions of all the joints throughout the body, and that the development of upright postural control involves the alteration of multi-joint coordination. Taken together, for a better understanding of developmental changes in upright postural control, it is important to establish alternative force platform measures that can easily evaluate the postural control strategy at the whole-body level. In the present study, we demonstrated that the COM_acc_ is a candidate for such measures.

### Limitations

In the present experiment, the participants were required to maintain only three 30-s-long periods of quiet standing for each visual condition. Although this experimental procedure was chosen because of the limited capacity of concentration in the children, it is possible that such a short length and small number of trials resulted in a poor estimate of the PSD, particularly at lower frequencies [36]. Next, our present approach provides only indirect evidence for differences in the multi-joint control of balance during quiet standing between young children and young adults. Therefore, further research comparing GRF to whole body kinematics/kinetics is necessary to obtain direct evidence. In addition, although the EC/EO ratios of COM_acc_ amplitudes indicate no difference in visual dependency, we cannot draw a conclusion about the visual dependency because of the substantial difference in COM_acc_ amplitude between age groups. Further studies are needed to address this issue. Finally, it should be kept in mind that the present study is a comparison study only between children 5–6 years old and young adults. Interestingly, some studies have reported nonlinear changes in upright postural control throughout development [6,7,37]. Therefore, to clarify the whole image of the developmental changes in upright postural control, it is still vital to investigate the postural control strategy in children of a wide range of ages, and at the same time to investigate the underlying neural mechanisms.

## Conclusions

In the present study, we demonstrated that both the COM_acc_ and COP can clearly distinguish the difference in postural control during quiet standing between young children and young adults and between eyes open and eyes closed conditions. We also found that the sway frequency of COM_acc_ in children was higher than that in adults, for which differences in biomechanical and/or neural factors between age groups may be responsible. The results suggest that the postural control strategy in children changes throughout development at the whole-body level. These results imply that the COM_acc_ can be an alternative force platform measure for assessing the developmental changes in upright postural control.

## Supporting Information

S1 Dataset Physical characteristics and measurement variables for all participants analyzed. (XLSX)

## References

[1] Peterka RJ, Black FO (1990) Age-related changes in human posture control: sensory organization tests. J Vestib Res 1: 73–85. 1670139

[2] Peterson ML, Christou E, Rosengren KS (2006) Children achieve adult-like sensory integration during stance at 12-years-old. Gait Posture 23: 455–463. 16002294 10.1016/j.gaitpost.2005.05.003

[3] Hsu Y-S, Kuan C-C, Young Y-H (2009) Assessing the development of balance function in children using stabilometry. International Journal of Pediatric Otorhinolaryngology 73: 737–740. 10.1016/j.ijporl.2009.01.016 19232750

[4] Riach CL, Hayes KC (1987) Maturation of postural sway in young children. Dev Med Child Neurol 29: 650–658. 3666328 10.1111/j.1469-8749.1987.tb08507.x

[5] Riach CL, Starkes JL (1994) Velocity of centre of pressure excursions as an indicator of postural control systems in children. Gait Posture 2: 167–172.

[6] Kirshenbaum N, Riach CL, Starkes JL (2001) Non-linear development of postural control and strategy use in young children: a longitudinal study. Exp Brain Res 140: 420–431. 11685395 10.1007/s002210100835

[7] Rival C, Ceyte H, Olivier I (2005) Developmental changes of static standing balance in children. Neurosci Lett 376: 133–136. 15698935 10.1016/j.neulet.2004.11.042

[8] Chen LC, Metcalfe JS, Chang TY, Jeka JJ, Clark JE (2008) The development of infant upright posture: sway less or sway differently? Exp Brain Res 186: 293–303. 18057920 10.1007/s00221-007-1236-1

[9] Ajrezo L, Wiener-Vacher S, Bucci MP (2013) Saccades improve postural control: a developmental study in normal children. PLoS One 8: e81066 10.1371/journal.pone.0081066 24278379 PMC3836891

[10] Winter DA, Patla AE, Prince F, Ishac M, Gielo-Perczak K (1998) Stiffness control of balance in quiet standing. J Neurophysiol 80: 1211–1221. 9744933 10.1152/jn.1998.80.3.1211

[11] Aramaki Y, Nozaki D, Masani K, Sato T, Nakazawa K, Yano H (2001) Reciprocal angular acceleration of the ankle and hip joints during quiet standing in humans. Exp Brain Res 136: 463–473. 11291727 10.1007/s002210000603

[12] Creath R, Kiemel T, Horak F, Peterka R, Jeka J (2005) A unified view of quiet and perturbed stance: simultaneous co-existing excitable modes. Neurosci Lett 377: 75–80. 15740840 10.1016/j.neulet.2004.11.071

[13] Sasagawa S, Ushiyama J, Kouzaki M, Kanehisa H (2009) Effect of the hip motion on the body kinematics in the sagittal plane during human quiet standing. Neurosci Lett 450: 27–31. 10.1016/j.neulet.2008.11.027 19027828

[14] Hsu WL, Scholz JP, Schoner G, Jeka JJ, Kiemel T (2007) Control and estimation of posture during quiet stance depends on multijoint coordination. J Neurophysiol 97: 3024–3035. 17314243 10.1152/jn.01142.2006

[15] Hodges PW, Gurfinkel VS, Brumagne S, Smith TC, Cordo PC (2002) Coexistence of stability and mobility in postural control: evidence from postural compensation for respiration. Exp Brain Res 144: 293–302. 12021811 10.1007/s00221-002-1040-x

[16] Pinter IJ, van Swigchem R, van Soest AJ, Rozendaal LA (2008) The dynamics of postural sway cannot be captured using a one-segment inverted pendulum model: a PCA on segment rotations during unperturbed stance. J Neurophysiol 100: 3197–3208. 10.1152/jn.01312.2007 18829852

[17] Yamamoto A, Sasagawa S, Oba N, Nakazawa K (2015) Behavioral effect of knee joint motion on body's center of mass during human quiet standing. Gait Posture 41: 291–294. 10.1016/j.gaitpost.2014.08.016 25248799

[18] Wu J, McKay S, Angulo-Barroso R (2009) Center of mass control and multi-segment coordination in children during quiet stance. Exp Brain Res 196: 329–339. 10.1007/s00221-009-1852-z 19484228

[19] Horak FB, Macpherson JM (1996) Postural orientation and equiliburium In: Rowell L, Shepherd J, editors. Handbook of physiology, Section 12: Exercise: Regulation and integration of multiple systems. New York, USA: Oxford University Press pp. 255–292.

[20] Masani K, Vette AH, Kouzaki M, Kanehisa H, Fukunaga T, Popovic MR (2007) Larger center of pressure minus center of gravity in the elderly induces larger body acceleration during quiet standing. Neurosci Lett 422: 202–206. 17611029 10.1016/j.neulet.2007.06.019

[21] Yu E, Abe M, Masani K, Kawashima N, Eto F, Haga N, et al (2008) Evaluation of postural control in quiet standing using center of mass acceleration: comparison among the young, the elderly, and people with stroke. Arch Phys Med Rehabil 89: 1133–1139. 10.1016/j.apmr.2007.10.047 18503811

[22] Kuo AD (1995) An optimal control model for analyzing human postural balance. IEEE Trans Biomed Eng 42: 87–101. 7851935 10.1109/10.362914

[23] Sasagawa S, Shinya M, Nakazawa K (2014) Interjoint dynamic interaction during constrained human quiet standing examined by induced acceleration analysis. J Neurophysiol 111: 313–322. 10.1152/jn.01082.2012 24089399

[24] Sasagawa S, Ushiyama J, Masani K, Kouzaki M, Kanehisa H (2009) Balance control under different passive contributions of the ankle extensors: quiet standing on inclined surfaces. Exp Brain Res 196: 537–544. 10.1007/s00221-009-1876-4 19506843

[25] Masani K, Popovic MR, Nakazawa K, Kouzaki M, Nozaki D (2003) Importance of body sway velocity information in controlling ankle extensor activities during quiet stance. J Neurophysiol 90: 3774–3782. 12944529 10.1152/jn.00730.2002

[26] Corriveau H, Hebert R, Raiche M, Prince F (2004) Evaluation of postural stability in the elderly with stroke. Arch Phys Med Rehabil 85: 1095–1101. 15241756 10.1016/j.apmr.2003.09.023

[27] Corriveau H, Prince F, Hebert R, Raiche M, Tessier D, Maheux P, et al (2000) Evaluation of postural stability in elderly with diabetic neuropathy. Diabetes Care 23: 1187–1191. 10937520 10.2337/diacare.23.8.1187

[28] Assaiante C, Amblard B (1992) Peripheral vision and age-related differences in dynamic balance. Hum Mov Sci 11: 533–548.

[29] Woollacott M, Debu B, Mowatt M (1987) Neuromuscular control of posture in the infant and child: is vision dominant? J Mot Behav 19: 167–186. 14988057 10.1080/00222895.1987.10735406

[30] Loram ID, Maganaris CN, Lakie M (2005) Human postural sway results from frequent, ballistic bias impulses by soleus and gastrocnemius. J Physiol 564: 295–311. 15661824 10.1113/jphysiol.2004.076307 PMC1456055

[31] Lakie M, Loram ID (2006) Manually controlled human balancing using visual, vestibular and proprioceptive senses involves a common, low frequency neural process. J Physiol 577: 403–416. 16959857 10.1113/jphysiol.2006.116772 PMC2000668

[32] Günther M, Müller O, Blickhan R (2011) Watching quiet human stance to shake off its straitjacket. Archive of Applied Mechanics 81: 283–302.

[33] Bernard-Demanze L, Dumitrescu M, Jimeno P, Borel L, Lacour M (2009) Age-related changes in posture control are differentially affected by postural and cognitive task complexity. Curr Aging Sci 2: 139–149. 20021408

[34] Kouzaki M, Masani K, Akima H, Shirasawa H, Fukuoka H, Kanehisa H, et al (2007) Effects of 20-day bed rest with and without strength training on postural sway during quiet standing. Acta Physiol (Oxf) 189: 279–292. 17305708 10.1111/j.1748-1716.2006.01642.x

[35] Scholz JP, Schoner G (1999) The uncontrolled manifold concept: identifying control variables for a functional task. Exp Brain Res 126: 289–306. 10382616 10.1007/s002210050738

[36] van der Kooij H, Campbell AD, Carpenter MG (2011) Sampling duration effects on centre of pressure descriptive measures. Gait Posture 34: 19–24. 10.1016/j.gaitpost.2011.02.025 21524912

[37] Riach CL, Starkes JL (1993) Stability limits of quiet standing postural control in children and adults. Gait Posture 1: 105–111.

